# Correlation between epicardial adipose tissue and myocardial injury in patients with COVID-19

**DOI:** 10.3389/fphys.2024.1368542

**Published:** 2024-04-19

**Authors:** Tianhong Su, Bincheng Zhong, Chao Tang, Shunsong Qiao, Yu Feng, Hao Peng, Xiaosong Gu

**Affiliations:** ^1^ Department of Cardiology, The Second Affiliated Hospital of Soochow University, Suzhou, Jiangsu, China; ^2^ Department of Emergency, The Tongren Hospital Affiliated to Shanghai Jiaotong University, School of Medicine, Shanghai, China; ^3^ Department of Endocrinology, The Second Affiliated Hospital of Soochow University, Suzhou, Jiangsu, China; ^4^ Department of Epidemiology, School of Public Health, Medical College of Soochow University, Suzhou, Jiangsu, China

**Keywords:** epicardial adipose tissue, COVID-19, myocardial injury, cTnI, LDL-C

## Abstract

**Background:** Many people infected with COVID-19 develop myocardial injury. Epicardial adipose tissue (EAT) is among the various risk factors contributing to coronary artery disease. However, its correlation with myocardial injury in patients diagnosed with COVID-19 remains uncertain.

**Methods**: We examined myocardial biomarkers in population affected by COVID-19 during the period from December 2022 to January 2023. The patients without myocardial injury were referred to as group A (*n* = 152) and those with myocardial injury were referred to as group B (*n* = 212).

**Results:** 1) The A group and the B group exhibitedstatistically significant differences in terms of age, TC, CRP, Cr, BUN, LDL-C, IL-6, BNP, LVEF and EAT (*p* < 0.05). 2) EAT volumehad a close relationship with IL-6, LDL-C, cTnI, and CRP (*p* < 0.05); the corresponding correlation coefficient values were 0.24, 0.21, 0.24, and 0.16. In contrast to those with lower EAT volume, more subjects with a higher volume of EAT had myocardial injury (*p* < 0.05). Regression analysis showed that EAT, LDL-C, Age and Cr were established as independent risk variables for myocardial injury in subjects affected by COVID-19. 3) In COVID-19 patients, the likelihood of myocardial injury rised notably as EAT levels increase (*p* < 0.001). Addition of EAT to the basic risk model for myocardial injury resulted in improved reclassification. (Net reclassification index: 58.17%, 95% CI: 38.35%, 77.99%, *p* < 0.001).

**Conclusion:** Patients suffering from COVID-19 with higher volume EAT was prone to follow myocardial injury and EAT was an independent predictor of heart damage in these individuals.

## 1 Introduction

Since March 2020, the COVID-19 pandemic has unfolded as a lethal viral crisis, unleashing widespread devastation on human lives and the global economy (An et al., 2022). The national healthcare systems are overwhelmed because COVID-19 cases often come with respiratory symptoms. Individuals with the virus infection present with a range of clinical symptoms, such as breathlessness, fever, fatigue, coughing, diarrhea, nausea, reduced lymphocytes, and some individuals may also experience neurological issues like headaches, dizziness, and changes in awareness levels. , and in severe cases, mortality can occur as a result of ARDS and low levels of oxygen saturation (Parasher, 2021).

Nevertheless, in clinical practice, the heart damage caused by COVID-19 is frequently disregarded or identified too late. A considerable proportion of patients experience myocardial injury are detected following their infection with COVID-19 (Abdi et al., 2022; Artico et al., 2023). It was shown that 32% of COVID-19 pneumonia patients experience acute injury to the heart muscle, which was observed through elevated levels of cTnI (Chen et al., 2021). Older patients with pre-existing medical conditions and those who have had COVID-19 are more susceptible to heart damage. This can lead to abnormal heart rhythms, heart attacks, and heart failure (Pellicori et al., 2021). COVID-19 patients with pneumonia and heart damage face a higher risk of experiencing severe medical condition, long recovery times, and increased chances of death for which many treatments are not very effective. Moreover, the exact understanding of the pathophysiological process by which COVID-19 causes damage to the heart muscle remains incomplete. As COVID-19 patients who also experience heart injury tend to have a negative prognosis (Al-Wahaibi et al., 2020), it is highly valuable to detect individuals who are prone to heart injury during their COVID-19 infection.

Epicardial adipose tissue (EAT) is a distinct fat deposit situated between the heart muscle and the inner layer of the heart’s outer covering. It has various impacts on cardiovascular disease. Localized inflammation is a direct result of EAT on coronary atherosclerosis (Karampetsou et al., 2022). EAT leads to myocarditis and harm to the heart by releasing substances that cause inflammation through the paravalvular wall (Lasbleiz et al., 2021). Additionally, the volume of epicardial fat can predict the severity of COVID-19 symptoms (Erdöl et al., 2022). It is possible that EAT could play a part in the cardiac syndrome associated with COVID-19 (Iacobellis, 2022). The reported diagnostic value of EAT for the severity and occurrence of heart damage in people who had a COVID-19 infection remains uncertain. Additional research is still required to examine the factors that contribute to the development of cardiac damage following infection with the novel coronavirus. In our study, we divided individuals with COVID-19 infection into groups with myocardial injury and groups without myocardial injury to study the role and significance of EAT in myocardial injury. This research will offer a fresh approach in diagnosing patients who have both myocardial injury and COVID-19, pointing them towards a new direction.

## 2 Study approach

### 2.1 Research sample

The individuals were adult patients diagnosed with COVID-19 by completing reverse transcription-polymerase chain reaction (RT-PCR) test and admitted to the Second Affiliated Hospital of Soochow University and the Tongren Hospital affiliated to Shanghai Jiao Tong University from Dec. 2022 to Jan. 2023, all of whom were tested for chest CT scan. The patients admitted to the hospital mainly have COVID-19 pneumonia according CT scan. We enrolled 364 COVID-19 patients without oxygen saturation decrease in our study. And it adhered to the STROBE checklist and obtained approval from the ethics board of the Second Affiliated Hospital of Soochow University (JD-HG-2023-76).

A total of 5 mL venous blood samples were collected from the study participants in the morning after a 12-h fasting period. After immediate centrifugation at 4°C, aliquots were stored at −80°C until analysis. FBG was measured by an automated glucose oxidase method (Automatic Analyzer 2700, Olympus, Tokyo, Japan). Serum total cholesterol (TC), triglyceride (TG), high-density lipoprotein cholesterol (HDL), low-density lipoprotein cholesterol (LDL-C), Cr, BUN, CRP, IL-6, BNP were measured by enzymatic methods. Troponin markers were evaluated within 24 h of hospital admission for all patients included in both cohorts to ensure consistency in the timing of exposure assessment. Tropoin I was measured using Troponin I (cTnI) detection kit, with each value reported as the mean of duplicate measurements made on the same serum sample. Test range was 0.01–50 ng/mL, and intraassay and interassay coefficient of variability (CVs) were less than 10% and 15%, respectively. The normal standard for cTnI with the kit is 0–30 ng/L.

Myocardial injury is defined as patients with cTnI elevated (Cao et al., 2020; Sandoval et al., 2020), and two cardiologists ruled out acute myocardial infarction based on clinical manifestations and electrocardiogram. According to whether cTnI levels exceed 30 ng/L, all participants are divided into two groups:group A comprised 152 cases of subjects who were not with myocardial injury, while 212 cases with myocardial injury were in group B. Apical two-chamber and four-chamber echocardiographic images were used to calculate volumes and left ventricular ejection fraction (LVEF) using Simpson’s biplane rule by investigators blinded to all patient data. In addition, Gender, BMI were recorded.

**FIGURE 1:**
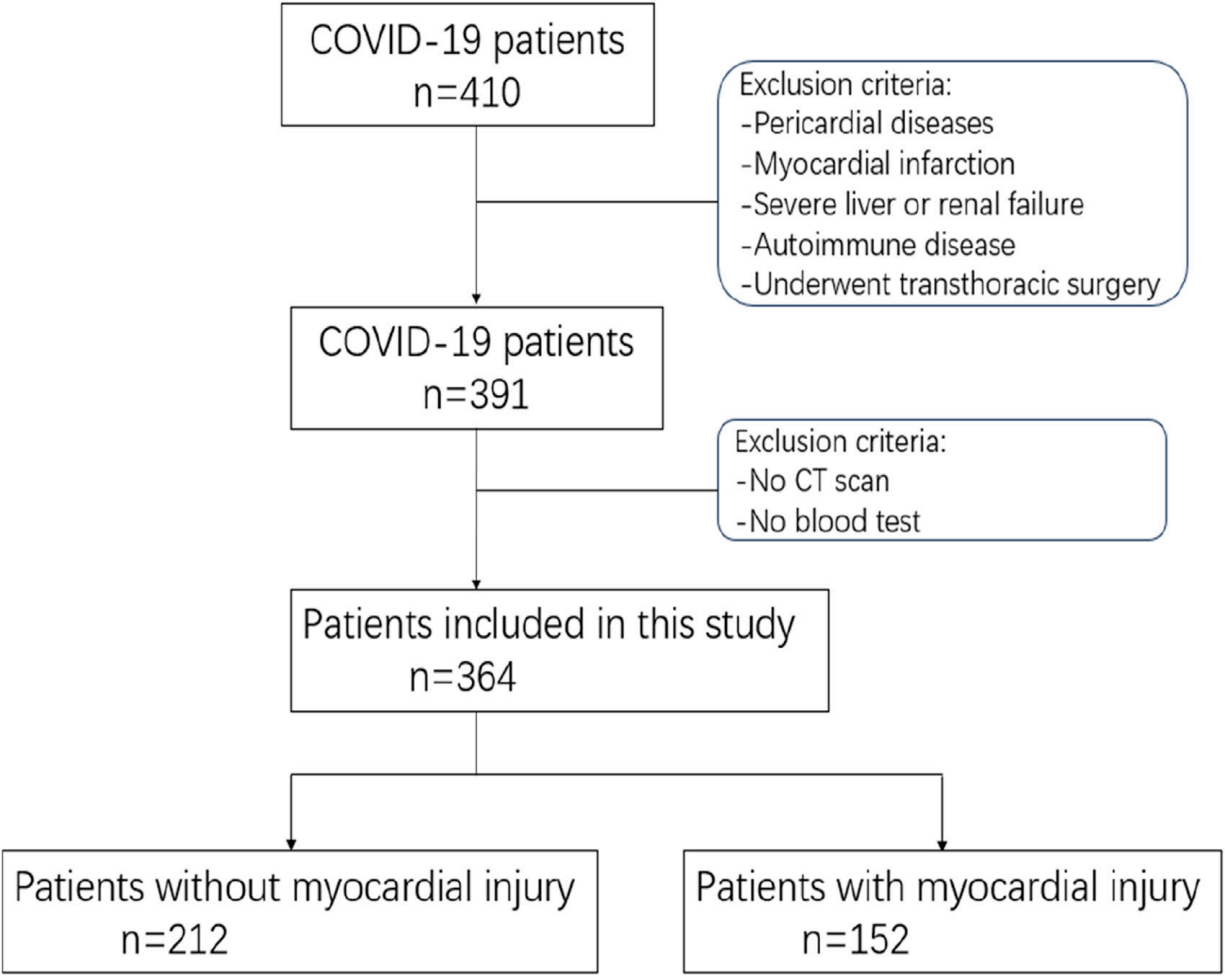
The process diagram depicting the participant pool illustrates the overall figures and rationales for exclusion at every stage.

Patients with following conditions were excluded. 1) Combination of severe cardiac emergencies, such as myocardial infarction, aortic dissection, etc., 2) Combination of severe heart valve diseases; 3) Combined with severe liver and kidney disfunction; 4) Combination of severe autoimmune diseases; 5) Combination of tumor and hematologic related diseases (Figure 1).

### 2.2 Assessment of EAT volume

**FIGURE 2:**
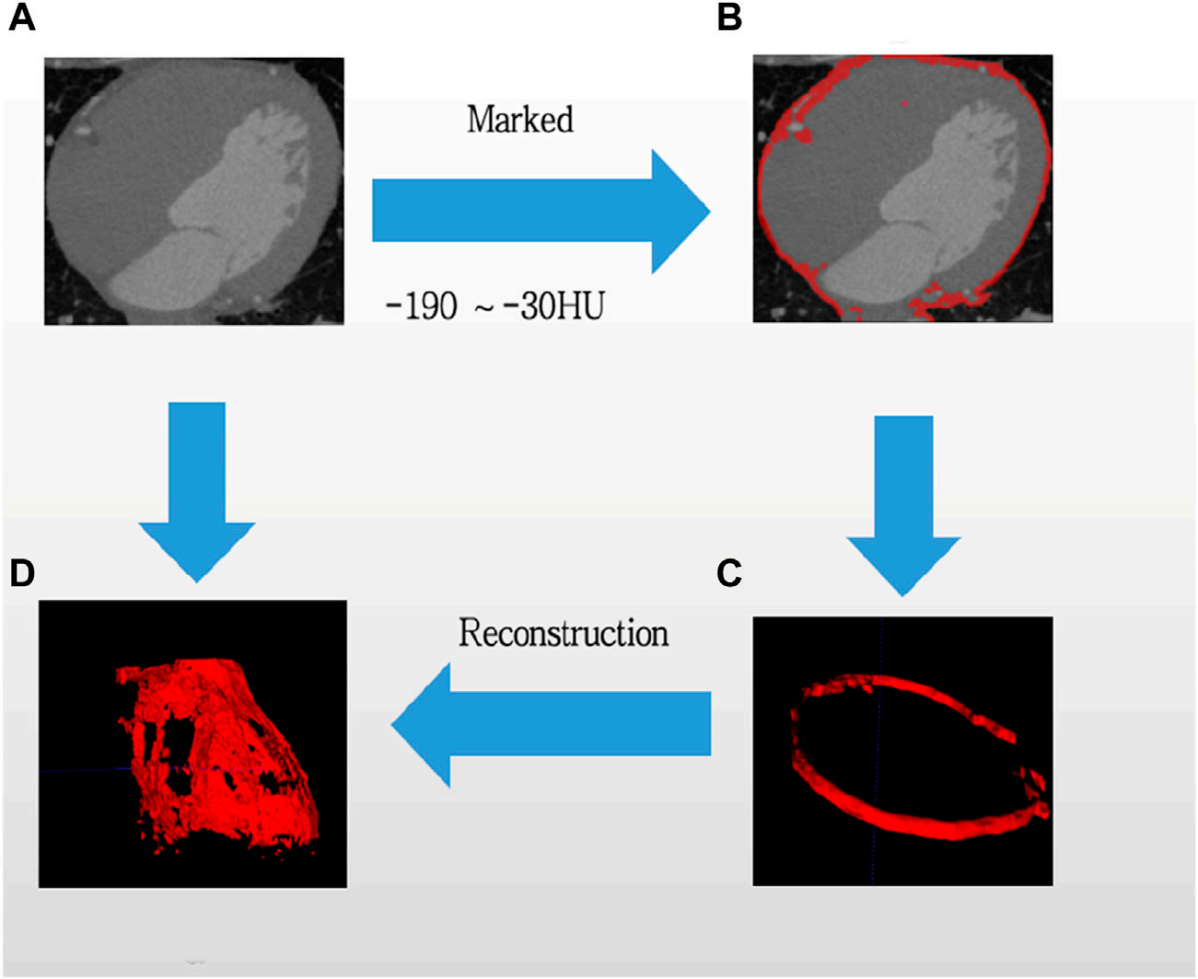
Computed tomography is utilized to evaluate epicardial adipose tissue. From original CT **(A)**, then selected the threshold value from −190HU to −30HU, and Marked the EAT tissue **(B)**, updated a flat EAT tissue **(C)**, finally reconstructed the images of EAT. Adipose tissue was emphasized in red, while the process was indicated by blue arrows. **(D)** Is reconstruction of EAT.

Epicardial adipose tissue volume was assessed by two clinicians who underwent specialized training in measuring EAT. In terms of chest CT, we chose electron beam CT scanning that would not need contrast agents, and we used GE (Healthcare, Milwaukee, United States) or Siemens (Healthiness, Erlangen, Germany) scanners to do the examinations. Tissue was classified as EAT if it fell within the parietal pericardial boundary and exhibited fat density attenuation on CT, with measurements ranging from −30 to −190 Hounsfield units (HU) (Xu et al., 2018). The superior and inferior boundary of the heart were determined as the pulmonic artery bifurcation and tip of the left ventricle, respectively. Right and left atrioventricular sulcus, anterior interventricular sulcus, and left atrioventricular sulcus were measured in the standard long-axis view of the heart, upper and lower interventricular sulcus were measured in short-axis views of the base of the heart, and finally in the right ventricular free wall (Xu et al., 2018). Epicardial fat pockets were manually outlined to determine their respective areas, and the cumulative epicardial fat area per slice was documented. Volumetric assessment was derived by the epicardial fat area in conjunction with a thickness of 3 mm (Figure 2).

### 2.3 Statistical methods

Utilizing the statistical program SPSS 26.0, the data were statistically evaluated. Continuous variables were presented as median [interquartile range (IQR)], categorical variables were presented as frequencies (percentages), and intergroup comparisons conducted the two independent sample *t*-test. The relationships between EAT levels and other variables were assessed using Pearson’s chi-squared test. Logistic regression was employed to analyze the association between EAT and myocardial injury in individuals. We utilized restricted cubic spline (RCS) analysis with 4 knots to examine the nonlinear correlation between increasing EAT and myocardial injury. Model comparisons were performed utilizing continuous and categorical net reclassification index (NRI) as well as integrated discrimination improvement (IDI). *p* < 0.05 (two-tailed) was considered meaningful. NRI indicated the improved accuracy in categorizing higher-risk patients into myocardial injurygroup and those who did not develop a heart damage into the lower-risk categories after incorporating EAT into prediction models. IDI measured the increased difference in mean probability of predicting myocardial injury risk in the cases compared to controls, indicating whether the addition of EAT improved the model’s ability to differentiate between cases and controls.

## 3 Results

### 3.1 Comparison of general data and biochemical indicators between the COVID-19 people with or without myocardial harm

In this research, 364 patients in total were analyzed, of whom 152 were in group without myocardial injury and 212 in group with myocardial injury according cTnI levels more than 30 ng/L. The two patient groups’ general information and biochemical indicators were compared, as indicated in Table 1. Compared to the without myocardial injury group, individuals within the myocardial injury group displayed a tendency towards older age, raised levels of Cr, BUN, CRP, LDL-C, IL-6, TC, BNP, and lower LVEF(*p* < 0.05). In addition, Gender, BMI, and TG levels found no notable discrepancies between the two cohorts statistically. (*p* > 0.05).

**TABLE 1:** Baseline comparison of study cohorts.

Variable	COVID-19 patients		*p*-value
	Without myocardial injury (*n* = 152)	With myocardial injury (*n* = 212)	
Gender, male	88 (58%)	135 (64%)	0.264
Age (y)	72 (66,78)	81 (73,87)	<0.001
BMI (kg/m^2^)	24.1 (21.5,26.5)	23.4 (20.7,25.7)	0.053
TC (mmol/L)	4.86 (4.14, 5.76)	5.33 (4.46, 5.99)	0.004
TG (mmol/L)	1.57 (1.11, 2.14)	1.65 (1.17, 2.40)	0.309
LDL-C (mmol/L)	2.65 (2.10, 3.36)	3.20 (2.50, 4.17)	<0.001
Cr (μmol/L)	69 (57, 84)	85 (65, 127)	<0.001
BUN (mmol/L)	5.8 (4.6, 7.6)	7.5 (5.4, 10.9)	<0.001
CRP (mg/L)	34 (13, 70)	58 (23, 87)	<0.001
IL-6 (pg/L)	14 (6, 42)	25 (10, 56)	<0.001
BNP (ng/L)	36 (18, 82)	147 (72, 364)	<0.001
LVEF (%)	64 (60, 68)	60 (55, 65)	<0.001
EAT (mm^3^)	88 (69, 130)	134 (89, 160)	<0.001
CTnI (ng/L)	0.01 (0.00, 0.01)	0.07 (0.03, 0.23)	<0.001

BMI, body mass index; CRP, C- reactive protein; Cr, creatinine; BUN, blood urea nitrogen; LDL-C, low-density lipoprotein cholesterol; BNP, brain natriuretic peptide; IL-6, interleukin-6; LVEF, left ventricular ejection fraction; EAT, epicardial adipose tissue; CTnI, cardiac troponin I; TG, triglycerides; TC, total cholesterol.

### 3.2 Correlation between EAT and clinical parameters

Within myocardial injury group, the correlation between the volume of EAT and other parameters in COVID-19 patients was analyzed. The findings revealed that the volume of EAT was positively associated with LDL-C, cTnI, CRP and IL-6 (correlation index: r = 0.21, 0.24, 0.16, and 0.24, respectively; *p* < 0.05), negatively relation with LVEF (r = −0.25, *p* = 0.024). The findings were displayed in Table 2.

**TABLE 2:** Correlation between EAT and clinical indicators in myocardial injury group (*n* = 212).

Variable	EAT	
	r	*p*-value
LVEF (%)	−0.25	0.024
LDL-C (mmol/L)	0.21	0.002
cTnI (0–30 ng/L)	0.24	0.000
CRP (mg/L)	0.16	0.022
IL-6 (pg/L)	0.24	0.000

LDL-C, low-density lipoprotein cholesterol; cTnl, cardiac troponin l; CRP, C- reactive protein; IL-6, interleukin-6; LVEF, left ventricular ejection fraction.

### 3.3 Univariate and multivariate logistic regression analyses risk of COVID-19 patients with myocardial injury

Univariate and multivariate logistic regression analyses for risk factors related to myocardial injury in the overall cohort are displayed in Table 3. The findings excluded the following variables as factors affecting myocardial injury- EAT (*p* trend < 0.001), age (*p* < 0.001), LDL-C (*p* < 0.001), CRP (*p* = 0.001), IL-6 (*p* = 0.009), TC (*p* = 0.007) and Cr (*p* < 0.001). The aforementioned variables were included in the multiple linear regression model, where it was observed that the following variables significantly contributed to myocardial injury—EAT (*p* trend < 0.001), Cr (*p* < 0.001), LDL-C (*p* = 0.003), and age (*p* < 0.001), as depicted in Table 3. OR (95% CI) associated with the third quartile of EAT was 4.10 (2.02–8.30) (*p* < 0.001).

**TABLE 3:** Univariate logistic regression analysis for COVID-19 with myocardial injury.

Variables	Univariate analysis		Multivariate analysis	
	OR (95% CI)	*p*-value	OR (95% CI)	*p*-value
Age (y)	1.07 (1.05–1.10)	*p* < 0.001	1.07 (1.05–1.10)	*p* < 0.001
CRP (mg/L)	1.01 (1.00–1.02)	*p* = 0.001	1.00 (1.00–1.01)	*p* = 0.327
IL-6 (pg/L)	1.01 (1.00–1.02)	*p* = 0.009	1.00 (0.99–1.01)	*p* = 0.759
Cr (μmol/L)	1.02 (1.01–1.02)	*p* < 0.001	1.01 (1.01–1.02)	*p* < 0.001
TC (mmol/L)	1.29 (1.08–1.55)	*p* = 0.007	1.09 (0.84–1.41)	*p* = 0.512
LDL-C (mmol/L)	1.83 (1.46–2.33)	*p* < 0.001	1.60 (1.17–2.18)	*p* = 0.003
Gender, male	1.28 (0.83–1.95)	*p* = 0.264		—
BMI (kg/m^2^)	0.96 (0.91–1.01)	*p* = 0.116		—
TG (mmol/L)	1.18 (0.96–1.46)	*p* = 0.120		—
BUN (mmol/L)	1.02 (0.99–1.05)	*p* = 0.230		—
EAT (mm3)		*p* trend <0.001		*p* trend <0.001
[∼,78]	—	—	—	—
[78,112]	1.55 (0.86–2.79)	*p* = 0.146	1.52 (0.78–2.99)	*p* = 0.223
[112,154]	4.68 (2.55–8.57)	*p* < 0.001	4.10 (2.02–8.30)	*p* < 0.001
[154,∼]	4.28 (2.26–8.10)	*p* < 0.001	3.24 (1.51–6.99)	*p* = 0.003

BMI, body mass index; CRP, C- reactive protein; Cr, creatinine; BUN, blood urea nitrogen; LDL-C, low-density lipoprotein cholesterol; IL-6, interleukin-6; EAT, epicardial adipose tissue; TG, triglycerides; TC, total cholesterol.

### 3.4 Receiver operating characteristic curves of EAT, LDL-C, Cr, and age were plotted, and area under curve (AUC) was calculated

**FIGURE 3:**
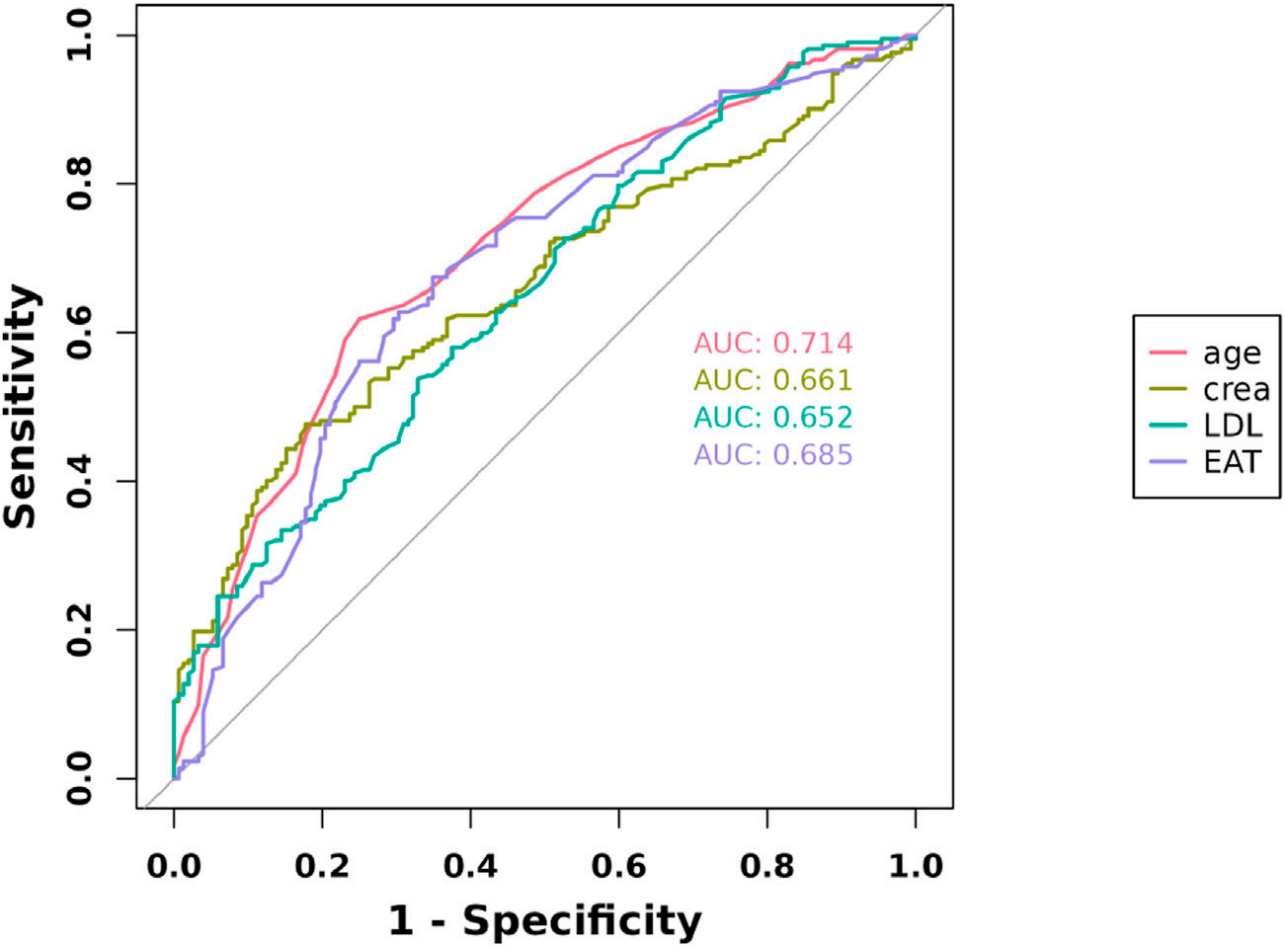
Receiver operating characteristic curves for myocardial injury estimated by the LDL-C, age, Cr, and EAT.

**FIGURE 4:**
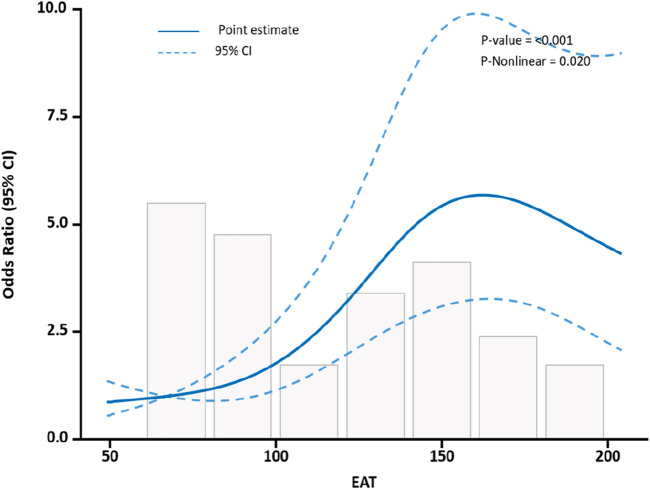
RCS to evaluate EAT levels with the risk of myocardial injury in subjects.

The ROC curves of the EAT, LDL-C, Cr, and age in the overall cohort are shown in Figure 3, and their AUCs were 0.685, 0.652, 0.66, and 0.714, respectively (Table 4). Restricted cubic splines were employed to assess the link between EAT and the risk of cardiac muscle damage. As depicted in Figure 4, a positive correlation between EAT and myocardial injury risk was identified (likelihood ratio test, p for non-linearity = 0.020). Knots were tested between 3 and 7, with the model featuring the lowest Akaike information criterion value chosen for RCS analysis. Finally, we used RCS with 4 knots. The reference value was set depending on the RCS shape).

**TABLE 4:** Area under the curve for various significant independent risk factors of myocardial injury.

Variables	AUC	Cutpoint	Youden index	Sensitivity	Specificity	PPV	NPV	Accuracy
Age	0.714	79	0.3679245	0.618	0.75	0.775	0.585	0.673
Cr	0.661	89.3	0.2987835	0.476	0.822	0.789	0.53	0.621
LDL	0.652	3.13	0.2087885	0.538	0.671	0.695	0.51	0.593
EAT	0.685	108	0.3258441	0.675	0.651	0.73	0.589	0.665

Cr, creatinine; LDL, low-density lipoprotein cholesterol; EAT, epicardial adipose tissue; PPV, positive predictive value; NPV, negative predictive value.

### 3.5 Enhanced discriminative capacity and reclassification were observed with the incorporation of EAT

We utilized clinical variables and univariate analysis to identify significant covariates, and subsequently evaluated whether EAT enhances the discrimination of COVID-19 myocardial injury compared to other risk factors. Initially, upon incorporating EAT into the baseline risk model consisting of age, Cr, and LDL-C, the c-index increased from 0.794 to 0.818 (*p* = 0.035) compared to the original model (Table 5). Furthermore, we examined the enhanced reclassification. Risk categories were delineated as low, medium, and high-risk, with thresholds set at 0.2 and 0.8, signifying probabilities of <20%, 20%–80%, and >80% for cardiac damage occurrence in subjects with the novel coronavirus. Finally, we obtained a continuous NRI (95% CI) of 58.17% (38.35%–77.99%) (*p* < 0.001), and a categorical NRI (95% CI) of 8.28% (1.19%–15.37%) (*p* = 0.022), indicating that incorporating EAT into the model increased the accurate reclassification percentage by 58.17% and improved the ordered categorical risk probability by 8.28%. It suggested that adding the predictive variable (EAT) improved the model’s accuracy in predicting myocardial injury. Additionally, we obtained an integrated discrimination improvement (IDI) of 3.94% (95% CI: 1.84%–6.03%), indicating that after incorporating additional factors—EAT, the model’s predictive ability increased by 3.94% (*p* < 0.001) (Table 5).

**TABLE 5:** Metrics for reclassification and discrimination of myocardial injury by EAT in subjects. Patients were divided into 3 risk categories: <20%, 20%–80%, >80%.

Model	C-index		Continuous NRI, %		Categorical NRI, %		IDI, %	
	Estimate (95% CI)	*p*-value	Estimate (95% CI)	*p*-value	Estimate (95% CI)	*p*-value	Estimate (95% CI)	*p*-value
Basic	0.794 (0.749–0.839)	0.035	Ref	<0.001	Ref	0.022	Ref	<0.001
Add EAT	0.818 (0.776–0.861)		58.17 (38.35–77.99)		8.28 (1.19–15.37)		3.94 (1.84–6.03)	

Basic: Model of age, Cr, LDL-C; NRI, net reclassification improvement; IDI, integrated discrimination improvement.

## 4 Discussion

Our research findings show that higher volumes of epicardial adipose tissue are linked to increased myocardial injury, as assessed by cTnI (Cao et al., 2020; Sandoval et al., 2020). EAT volume has a positive correlation observed with the parameters of CRP and LDL-C in our COVID-19 subjects, which agrees with observations from previous studies of coronary heart disease populations (Sever et al., 2013; Packer, 2018). However, it is noteworthy that EAT independently contributes to the risk of myocardial harm in individuals with COVID-19. EAT also serves as a predictor of cardiac tissue damage among subjects.

Since COVID-19 became popular worldwide in March 2020, many studies have focused on the multiple organ complications caused by it, such as acute myocardial injury, renal failure or thromboembolic events (Salyer et al., 2021; Ahamed and Laurence, 2022). COVID-19 poses potential implications for the cardiovascular health around the globe. The virus has the capability to affect not only the lungs but also the heart, vascular tissues, and circulating cells. The occurrence of acute cardiac muscle damage is increasingly recognized as a common extrapulmonary complication of the novel coronavirus, carrying potential long-lasting implications (Chung et al., 2021). Considering the significant inflammatory nature of COVID-19, COVID-19 survivors may be at risk of developing persistent residual myocardial injury (Shchendrygina et al., 2021). Our study also finds that the population of patients with myocardial injury has higher inflammatory indicators.

Numerous studies have delved into the potential mechanisms underlying myocardial injury induced by COVID-19 (Babapoor-Farrokhran et al., 2020). Vascular endothelial cells are pivotal in maintaining vascular homeostasis and regulating the coagulation system. In a healthy state, endothelial cells express factors that promote vascular relaxation, enhance blood flow, inhibit platelet aggregation and coagulation, and facilitate fibrinolysis. However, dysfunctional endothelial cells disrupt this balance, leading to vascular constriction and thrombus formation (Broekman et al., 1991). Infection with COVID-19 may directly lead to endothelial injury and dysfunction. Examinations of autopsies and biomarkers indicate that endotheliitis plays a role in causing acute respiratory distress syndrome (ARDS) in patients with the novel coronavirus. SARS-CoV-2 infects and disrupts the functions of lung endothelial cells (Joffre et al., 2022). Endothelial dysfunction linked to COVID-19 can initiate organ impairment and thrombosis occurrences (Gu et al., 2021). Abnormal endothelial function and thrombus formation are important mechanisms of myocardial injury, therefore, the mechanism of myocardial injury caused by COVID-19 may be caused by endothelial dysfunction and thrombosis.

While the heart and blood vessels are susceptible to novel coronavirus, there is limited substantial evidence supporting direct myocardial infection by SARS-CoV-2. Further pathological investigations and autopsy analyses are essential for elucidating the virus’s potential to directly induce myocarditis (Halushka and Vander Heide, 2021). Endomyocardial biopsy conducted on five patients demonstrate heightened macrophage counts in all cases, with one patient showing signs of lymphocytic myocarditis. Notably, qRT-PCR do not identify any SARS-CoV-2 RNA in any of the biopsies (Weckbach et al., 2021).

Inflammation increase is involved in cardiac injury was reports by many studies (Patel et al., 2022). Histologically, no COVID-19 samples have been detected as viral myocarditis. However, as time progresses, quantitative analysis using MPX reveals a notable rise in the quantity of perivascular CD11b/TIE2+ macrophages in the cases. This indicates that the development of heart damage in the individuals with the virus is primarily driven by a macrophage-driven perivascular inflammatory process (Werlein et al., 2023). Patients experiencing severe complications have higher C-reactive protein and troponin T level, the severity of cardiovascular complications like heart rate variability and heart failure following virus infection associated with elevated CRP (Lewek et al., 2021; Patel et al., 2022). Our research also finds that IL-6 and CRP is significantly increased, suggesting that inflammation may be one of the mechanisms contributing to myocardial harm in cases of COVID-19.

Elevated lipid levels may exacerbate the prognosis of individuals with the novel coronavirus. The research indicates that the ratios of TG/HDL-C, TC/HDL-C, LDL-C/HDL-C, and the TG index exhibit statistical correlations with death rate from the coronavirus disease 2019 (Rohani-Rasaf et al., 2022). Moreover, individuals with heterozygous familial hypercholesterolemia face heightened risks of cardiovascular complications in the acute stage of COVID-19 infection. During the heightened inflammatory response in COVID-19 pneumonia, elevated LDL-C levels can exacerbate endothelial dysfunction and promote the release of prothrombotic mediators (Vuorio et al., 2023). Another study indicates that patients who has minimal LDL-C upon admission are more prone to immune and inflammation dysfunction, as well as multi-organ dysfunction (Zhao et al., 2021). Additionally, Gong et al. identified a U-shaped curve relationship between LDL-C levels and the risk of severe COVID-19, both diminished and heightened LDL-C levels were significantly linked to a greater risk of severe COVID-19 outcomes (Gong et al., 2021). In conclusion, dyslipidemia may be closely related to the prognosis of COVID-19, but there is currently no study exploring the clinical correlation between LDL-C and myocardial injury. Our research has made a valuable discovery that increased blood lipid level is one of the elements contributing to COVID-19-related myocardial injury.

Numerous studies highlight the close correlation between epicardial adipose tissue (EAT) and the severity of COVID-19-related illnesses (Mehta et al., 2022). EAT attenuation independently predicted critical illness respect to time to death or admission to ICU (Conte et al., 2021). EAT volume shows associations with inflammatory parameters, while EAT area serves as a predictive marker for mortality among young adult patients (Malavazos et al., 2020; Emekli et al., 2022), and an EAT volume of 97 cm³ demonstrates strong sensitivity and specificity in predicting a more extensive pulmonary involvement and consequently, a poorer clinical outcome in patients with SARS-CoV-2 pneumonia (Marcucci et al., 2022). Additionally, irrespective of BMI, age, and comorbidities, there is a notable correlation between EAT thickness and both the severity and mortality of COVID-19, Notably, heightened EAT thickness is linked to elevated levels of ultrasensitive cardiac troponins (Börekçi et al., 2014). Our research also found that in patients suffering from COVID-19, EAT volume was related to the degree of pneumonia, and the CUT OFF value of EAT volume had a very valuable predictive significance for myocardial injury among these patients. We did not investigate the mechanism between EAT and myocardial injury. The underlying mechanism may be as follows, firstly, high levels of EAT have a pro-inflammatory effect, and EAT patients may cause myocardial damage through inflammatory mechanisms (Malavazos et al., 2020). Secondly, high EAT is related to LDL-C, and LDL-C also plays a significant role in COVID-19 related myocardial injury (Gong et al., 2021), Therefore, high levels of EAT leads to myocardial injury may be associated with high levels of LDL-C. Finally, high EAT is associated with coronary microcirculation disorders (Nakanishi et al., 2017), while COVID patients usually have a hypercoagulable state (Levi et al., 2020). So high EAT patients are prone to form micro thrombosis and lead to myocardial damage.

The observations of the current investigation are limited by the small number of patients. Therefore, the clinical implementation of the results may be restricted. Further studies with many patients are required to confirm our results. Secondly, as this is a cross-sectional study, the causal relationship between EAT and myocardial injury needs to be confirmed by longitudinal and/or interventional studies. Thirdly, our study does not further analyze the duration of patients infected with COVID-19, so it may have a certain impact on inflammation and the quantification of myocardial enzymes.

## Data Availability

The raw data supporting the conclusion of this article will be made available by the authors, without undue reservation.
